# Tumor Suppressor Genes within Common Fragile Sites Are Active Players in the DNA Damage Response

**DOI:** 10.1371/journal.pgen.1006436

**Published:** 2016-12-15

**Authors:** Idit Hazan, Thomas G. Hofmann, Rami I. Aqeilan

**Affiliations:** 1 Lautenberg Center for Immunology and Cancer Research, IMRIC, Hebrew University-Hadassah Medical School, Jerusalem, Israel; 2 Cellular Senescence Group, Department of Epigenetics, German Cancer Research Center (DKFZ), Heidelberg, Germany; 3 Department of Cancer Biology and Genetics, The Ohio State University Wexner Medical Center, Columbus, Ohio, United States of America; 4 Department of Biochemistry, University of Vermont College of Medicine, Burlington, Vermont, United States of America; University of Washington School of Medicine, UNITED STATES

## Abstract

The role of common fragile sites (CFSs) in cancer remains controversial. Two main views dominate the discussion: one suggests that CFS loci are hotspots of genomic instability leading to inactivation of genes encoded within them, while the other view proposes that CFSs are functional units and that loss of the encoded genes confers selective pressure, leading to cancer development. The latter view is supported by emerging evidence showing that expression of a given CFS is associated with genome integrity and that inactivation of CFS-resident tumor suppressor genes leads to dysregulation of the DNA damage response (DDR) and increased genomic instability. These two viewpoints of CFS function are not mutually exclusive but rather coexist; when breaks at CFSs are not repaired accurately, this can lead to deletions by which cells acquire growth advantage because of loss of tumor suppressor activities. Here, we review recent advances linking some CFS gene products with the DDR, genomic instability, and carcinogenesis and discuss how their inactivation might represent a selective advantage for cancer cells.

## Introduction

Common fragile sites (CFSs) are genomic regions that display breaks, gaps, and constrictions in metaphase chromosomes grown under conditions that impede DNA synthesis, a state known as replication stress (Box 1) [1–3]. Numerous studies demonstrate that replication stress is a major cause of genome instability both in preneoplastic and neoplastic lesions (reviewed in [3, 4]). The current DNA damage model for cancer development suggests that oncogene activation results in replication stress preferentially at CFSs [5]. The breaks at CFSs occur likely because of collisions between replication forks and transcription machinery since CFSs tend to replicate relatively late during S phase and frequently associate with large (>1 Mb) genes ([6] and Box 2). These breaks at CFSs render them prone to copy number variations (CNVs) [7] resulting in gross chromosomal arrangements such as translocations, deletions, and amplifications as well as DNA double strand breaks (DSBs) associated with hypermutation that are locus- and cell type–specific [5, 8, 9]. Therefore, CFSs, which were historically mapped for the first time in lymphoblasts, are largely considered as hotspots for genomic instability [8–10] and preferential hotspots for viral DNA integrations [11, 12]. Since many of the focal deletions observed in CFSs using CNVs studies are hemizygous, these aberrations were proposed to be passenger mutations [13–15]. Altogether, these observations and others led to the view that CFS loci are inert fragile structures and their expression (induction) in cancer does not confer a growth advantage [15]. However, these observations still raise the question of why CFSs were not eliminated during evolution? The facts that CFSs remained conserved and tend to be deleted rather than amplified in cancer [13, 14] suggest that these regions and the encoded genes are not just passive “hotspots” for genomic instability but may play a central role in suppression of cancer initiation and/or progression. Indeed, many of the CFSs harbor functional genes that challenge the claim that these sites are merely inert structures. In fact, knockout mouse models for some CFS-gene products (CFS-Ps) revealed increased tumor burden [16, 17], suggesting growth advantage upon loss of these products. Hemizygous deletions are frequent in CFS genes (CFS-Gs), suggesting that these genes are haploinsufficient [16, 17] or that the second allele could be epigenetically silenced, as is seen for many CFS-Gs [18–22]. Furthermore, emerging findings show that CFS-Ps are directly involved in the DNA damage response (DDR) and therefore participate in maintaining genomic integrity (reviewed in [23–25]). These studies reveal that expression and activity of several CFS-Ps are induced in response to DNA damage and contribute to proper cellular checkpoint response, including DNA repair, cell cycle arrest, and apoptosis ([24, 26] and sections below). Impaired function of these checkpoints following deletion of CFS-Gs is likely to contribute to increased mutagenesis and genomic instability (discussed below). The view that at least some CFS-Ps are playing a driver role in cancer (and possibly other diseases) is gaining more ground and hence will be discussed in here.

**Box 1. Historical Overview of Fragile Sites**

CFSs were defined by Glover and colleagues in 1984 as ‘‘hot spots” for breaks and gaps induced by aphidicolin, a specific inhibitor of DNA polymerases α, δ, and ε leading to replicative stress and chromosomal aberrations [1]. The first observation of a fragile site was reported by Anatole Dekaban in 1965 when he observed a break in chromosome 9 in a blood sample isolated from a woman with recurrent eczematous dermatitis, for which she had received repeated X-ray treatment [27]. The break was seen in blood lymphocytes but not in a skin sample, implying the cell type–specific fragility. Later, fragile sites were classified into rare or common subgroups according to their frequency in the population, though they differ in other features as well [28]. Rare fragile sites are seen in less than 5% of the population, inherited in a Mendelian manner, and are associated with human genetic disorders such as fragile X syndrome. In contrast, CFSs represent the largest class of fragile sites present in all individuals [2]. In some tumors, CFSs were shown to be a preferential site for viral integration and are frequently associated with these cancers [25]. CFSs are differentially expressed between cell types and depend on the specific stress or DNA-damage agent [29–32]. Therefore, although currently around 90 CFSs have been identified in the human genome, the exact number differs depending on cell type, damage source, and method of analysis.

More recently identified types of fragile sites are early replicating fragile sites (ERFSs) which resemble recurrent, early replicating, and activation-induced cytidine deaminase (AID)-independent DNA lesions. ERFSs were observed in replicating B lymphocytes colocalized with highly expressed gene clusters and are enriched for repetitive elements and CpG dinucleotides. Intriguingly, more than 50% of recurrent amplifications and/or deletions in human diffuse large B cell lymphoma map to ERFSs [33].

**Box 2. Mechanisms of CFS Instability**

The working model of CFS expression posits that these regions are late replicating and present a challenge for DNA polymerases. Emerging evidence assessing breaks at CFS loci revealed that endonuclease MUS81–EME1 resolves incompletely replicated intermediates—preventing uncontrolled chromosome breakage at CFSs—and promotes genome integrity as a mean of genetic surveillance mechanism [34, 35]. If these unreplicated regions are not resolved (repaired) and are allowed to persist into mitosis, chromosomes could break, leading to sister chromatid exchanges, deletions, and translocations as observed in cancer cells. Indeed, defective DNA replication or failure to restart stalled replication forks appears to be a major risk factor for chromosomal abnormalities. CFSs are hence considered as hotspots for replication stress, and loss of CFS heterozygosity has emerged as a signature of stalled replication forks [36]. The sensitivity of CFSs to replication stress could stem from multiple factors, including: (i) formation of DNA secondary structures within CFSs, (ii) inability to recover and restart stalled forks, (iii) paucity of replication origins within CFS loci, and (iv) replication transcription interference specifically at large genes (>1 Mb).

Recent studies strongly support a significant role for replication–transcription interference. Many CFSs contain very large genes that are transcribed late in cell cycle and are targets of alterations in multiple cancers (reviewed in [25]). While collisions between transcription and replication can lead to genomic instability, eukaryotic cells appear to largely avoid this problem by segregating these two processes within spatially and temporally separated domains [37]. However, Helmrich and colleagues have found that the unique features of CFS and their associated large genes can cause collisions between replication forks and transcription machinery, leading to DNA breaks at these loci [6]. More recently, Wilson and colleagues [7] showed that, within specific cell lines, CFSs coincided with hotspots for copy number variants (CNVs). Further, they discovered that these regions of CFS and CNV hotspot overlap were robustly associated with very large (>1 Mb) active transcription units. They concluded that under replication stress, these active large transcription units drive extreme genomic instability that is locus- and cell type–specific. This disruption in replication dynamics produces both CNVs and CFSs.

Further characterization of fragile sites, their gene products, and their mechanisms of instability would shed light on their role in homeostasis and human pathologies. It is quite intriguing that recent evidence links controlled breaks with tissue homeostasis—i.e., brain activity [38–40]. The potential functional significance of DSBs and CFS-encoded genes in this context has not yet been determined and should have great implications.

For a more detailed introduction to the mechanisms of CFS instability, we recommend some recent reviews on this topic [2, 41, 42].

## CFSs: Accumulation of DSBs, Colocation with Tumor Suppressor Genes, and Association with Specific Cancer Types

**Fig 1:**
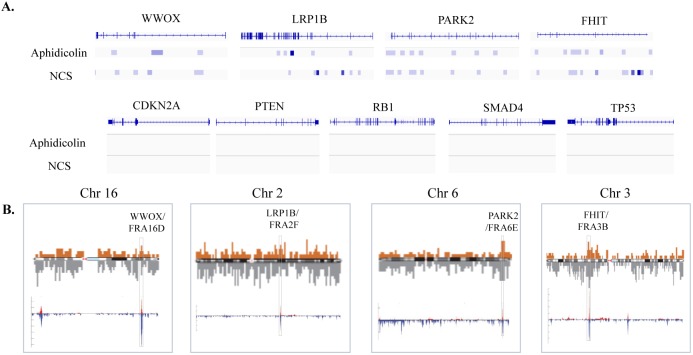
The sensitivity of CFS tumor-suppressor genes to DSBs as compared to their deletions in cancers. **A**. DSBs occur at CFS-Gs but not at classical tumor suppressors in HeLa cells treated with aphidicolin and neocarzinostatin (NCS) by BLESS (re-analyzed from [44]). **B**. Chromosome-wide mapping of DSBs in HeLa cells treated with aphidicolin (brown) and NCS (grey) by BLESS [44] compared to the genomic deletion profile in 746 cancer cell lines [13]. Homozygous (red) and hemizygous (blue) deletion. The rectangles mark loci of the CFS-Gs.

The prevalence of genetic alterations (mostly deletions) at CFSs could stem from their sensitive structure (Boxes 1 and 2). However, whether these aberrations confer a selective advantage in cancer development is poorly studied. Recent breakthroughs in next*-*generation sequencing techniques have provided new evidence that deletions of CFSs are significantly enriched in cancer, indicating that their loss may play a central role in cancer initiation and/or progression [13, 14]. Direct in situ Breaks Labeling, Enrichment on Strepavidin, and next-generation Sequencing (BLESS) is a new method, developed by Crosseto and colleagues, to map DSBs at a nucleotide resolution across the whole genome [43]. Using BLESS on HeLa cells treated with aphidicolin or neocarzinostatin, revealed accumulation of breaks at known CFSs that localize to apparent tumor suppressor genes: *FHIT/FRA3B*, *WWOX/FRA16D*, *LRP1B/FRA2*, and *PARK2/FRA6E* (Fig 1A). Interestingly, the BLESS analysis also showed that well-known classical tumor suppressors such as *CDKN2A*, *RB1*, *PTEN*, and *SMAD4*, although significantly deleted in cancers, were not enriched for DSBs [43]. The resistance of accumulating breaks in "classical tumor suppressors" compared to the sensitivity of CFS-related tumor suppressor genes implies an alternative mechanism for their inactivation. When comparing the distribution of DSBs and the genomic deletion profile of 746 cancer cell lines (from [13]), we found that CFS-Gs are more susceptible to breaks (Fig 1B, upper panel), and their loss (through deletions) is significantly enriched by selective pressure during cancer progression (Fig 1B, lower panel).

**Fig 2:**
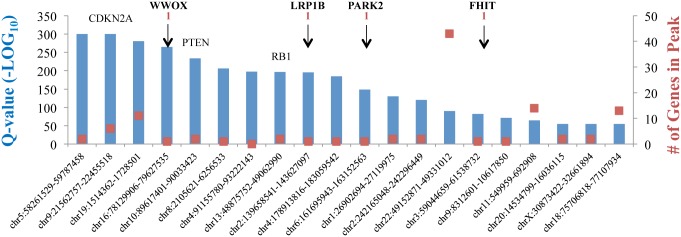
Most frequently deleted genomic loci in 8,822 epithelial cancer samples from 25 cancer types. Data contains copy number data from non-TCGA projects [14] and data generated by the Broad Institute TCGA Genome Characterization Center of the GISTIC portal. The blue bars (left *y-*axis) are Q-values (–Log10), representing that the gene inside the locus significantly enriched for deletion because of selective pressures. Highly deleted genes imply their role as tumor suppressors. The red dots (right *y*-axis) are the number of genes inside the peak. Numbers under CFS genes emphasize that these are unique within their peak.

Further support is supplied by data obtained from the TCGA copy number portal GISTIC (http://www.broadinstitute.org/tcga/home), which show that some CFS-Gs are significantly deleted in 8,822 samples of 25 different epithelial cancers types (Fig 2) while none were amplified. These observations argue that CFS-Ps might function as oncosuppressors. According to that analysis, loss of a given gene is considered to be a “driver” of cancer when it is significantly deleted (Q-value < 0.25) and present within the deleted peak since it suggests enrichment by selective pressures. Q-values determined by reanalysis of the GISTIC database identified several CFS-Gs in this category, including *WWOX* (1.24E^-265^), *LRP1B* (1.85E^-196^), *PARK2* (1.02E^-149^), and *FHIT* (8.70E^-72^), emphasizing their significant roles as tumor suppressors. These Q-values are within the range or even higher than some well-known tumor suppressors, including *RB1* (3.29E^-234^), *PTEN* (1.29E^-197^), and *SMAD4* (7.22E^-23^). Other CFS-Gs are either outside the peak or not significantly deleted in epithelial cancers. Additional analyses yielded Q-values demonstrating that some of these other CFS-Gs are significantly deleted in specific cancers—e.g., *GRID2* in uterine corpus endometrioid carcinoma (Q-value = 8.28E^-05^) and *IL1RAPL1* in head and neck squamous-cell carcinoma (Q-value = 1.19E^-12^). These data further confirm that the expression of CFSs in cancer is cell type–specific, in agreement with recent reports [29–32]. Future analysis should therefore consider all other CFS-Gs in a tissue-dependent manner.

## The DNA Damage Response (DDR) Is Associated with Replication Stress

Cells are constantly exposed to endogenous and exogenous DNA damaging agents, which may lead to chromosomal abnormalities [45]. Insufficiency of nucleotide biosynthesis has been also associated with replication stress, the source of CFS expression, and genomic instability [46]. DNA repair through the DDR is therefore critical for maintaining genome stability. The DDR is a complex signaling network that locates and orchestrates biological responses to DNA damage, including cell cycle arrest, repair of DNA lesions, apoptosis, and senescence [47, 48]. The ability to experimentally induce DSBs with ionizing radiation or chemotherapeutic drugs and ssDNA breaks (which may stall replication forks) with aphidicolin or hydroxyurea has allowed delineation of the major actors in the DDR signaling cascade. Both DNA lesions activate the master checkpoint kinases: DSBs activate ataxia telangiectasia mutated (ATM) while DNA single strand breaks (SSBs)—triggered by, for example, replicative stress—activate ataxia telangiectasia and Rad3–related kinase (ATR) [47, 48]. Activated ATM or ATR phosphorylate targets that transmit the signal to effectors that execute either repair, cell cycle arrest, apoptosis, or senescence. Defects in DDR signaling or repair contribute to various disorders and diseases, including developmental defects, neurodegenerative diseases, and cancer [49, 50].

Two groundbreaking studies proposed a hierarchical model for replication stress, DDR, and CFSs during cancer progression [8, 9], suggesting that an aberrant proliferation in preneoplastic lesions leads to DNA replication stress. This replication stress, either directly or through the formation of DSBs, can activate the ATR or ATM DNA-damage checkpoints. At that point, the cells either arrests in the cell cycle until the damage is repaired, or if the damage is unrepairable, cells undergo apoptosis or senescence [51]. Since DSBs preferentially occur at CFSs upon replication stress, these sites are prone to allelic imbalances and loss of heterozygosity (LOH) [10]. At later stages in cancer progression, telomere attrition and hypoxia also contribute to checkpoint activation and genomic instability. Eventually, tumor-suppressor loci (such as *p53*) are targeted, releasing cells from the suppressive effects of the DNA-damage checkpoint pathway and facilitating tumor progression [8, 9]. Analysis of SNP array data revealed that at early stages of cancer evolution, LOH occurs at CFSs with high frequency, while in more advanced tumors this rate decreases because of widespread genomic instability throughout the genome [10]. This difference may derive from progressive loss of CFS-P tumor-suppressor activities, which might provide a direct growth advantage, as well as from the loss of other DDR checkpoint proteins. Consistent with this hypothesis, emerging evidence implicates a number of CFS-Ps in maintenance of the DDR, cell cycle checkpoint, and genome stability (see below).

## CFS Gene Products with Roles in the DDR

Numerous animal model studies have reported that depletion of CFS-Gs correlates with initiation and progression of different types of cancer [16, 17, 52–58], suggesting that their encoded CFS-Ps act as tumor suppressors. These studies provide evidence for the mechanisms underlying their tumor-suppressing functions, in particular in response to DNA damage. Here, we discuss recent advances in knowledge about the role of specific CFS-Ps in the regulation of the DDR and tumor suppression.

### FRA3B/FHIT

The fragile histidine triad (*FHIT*) gene spans FRA3B at chromosome 3p14, which is the most fragile locus in human lymphoblasts [30–32]. Deletions, loss of expression, and other alterations of the *FHIT* locus have been frequently observed in a variety of cancers [59–62] and in premalignant lesions [63, 64]. *Fhit* knockout mouse models are much more susceptible to develop both spontaneous and carcinogen-induced tumors than wild-type control mice [16, 52]. As a tumor suppressor, FHIT’s role in the DDR is mainly guarding genome integrity and regulating apoptosis (reviewed in [23]). Pioneering work by the Huebner group showed that FHIT protects genomic integrity by preventing spontaneous replication stress. In FHIT-deficient cells, replication stress is caused by nucleotide imbalance associated with a decrease in thymidine triphosphate pools [65]. As a result, FHIT-deficient cells accumulate stalled replication forks and spontaneous DSBs, resulting in chromosome aberrations and acquisition of cancerous phenotypes. These data suggest that loss of FHIT expression in precancerous lesions may initiate genomic instability, linking alterations at CFSs to the origin of genome instability. In a recent report, the Huebner group analyzed whole exome sequences of *Fhit*-deficient tissues and cells and compared them to a control C57Bl6 genome, identifying hundreds of single-base substitutions associated with FHIT deficiency. The mutation signature is characterized by increased C>T and T>C mutations, in accordance with the signature identified in human malignancies [66].

FHIT is also involved in apoptosis through additional mechanisms: (i) in response to oxidative stress, FHIT interacts with ferrodoxin reductase (Fdxr) in mitochondria to enhance reactive oxygen species (ROS) production followed by caspase-3 activation and thus apoptosis [67–70]; (ii) in lung cancer cells treated with the chemotherapeutic drug paclitaxel, FHIT mediates apoptosis by preventing annexin 4 translocation from the cytosol to the plasma membrane [71]; (iii) another FHIT-mediated apoptosis mechanism involves release of Ca^2+^ from the mitochondrial stores, which leads to cell death [70]. Of note, these tumor suppressive functions of FHIT are mediated by its interacting partners rather than by its intrinsic catalytic hydrolase and/or transferase activity [72]. The highly conserved tyrosine 114 (Y114) within the HIT domain of FHIT can be phosphorylated by Src [73, 74] and has been shown to trigger caspase-dependent FHIT-mediated apoptosis [75]. A recent report demonstrated that FHIT regulates microRNA expression and modulates tumor progression by controlling epithelial–mesenchymal transition genes [76].

Further support for the role of FHIT in the DDR comes from DNA methylome analysis showing that epigenetic silencing of FHIT as a determining factor for radiosensitivity in oral cancer [77]. Ectopic expression of FHIT inhibited tumor growth in both in vitro and in vivo models and resensitized radioresistant cancer cells to irradiation by restoring Chk2 phosphorylation and G2/M arrest [77].

Taken together, these findings reveal a multifaceted role for FHIT as a tumor suppressor, including multiple functions in the DDR, defining it as an important guardian of the preneoplastic genome.

### WWOX/FRA16D

WW domain–containing oxidoreductase (*WWOX*) is a tumor suppressor gene that spans FRA16D at 16q23. Loss of WWOX expression because of gene deletions, loss of heterozygosity, chromosomal translocations, or epigenetic silencing is associated with poor prognosis in numerous types of cancer (reviewed in [78]). This region is also involved in a translocation at (14q32;16q23) observed in up to 25% of multiple myelomas [79]. Reestablishment of WWOX expression in WWOX-deficient, cancer-derived cell lines resulted in growth inhibition, apoptosis, and eventual inhibition of tumorigenicity [55, 80–82], suggesting its tumor suppressor function.

*WWOX* encodes a 46-kDa protein that contains two WW domains (WW1+2) and a short-chain dehydrogenase/reductase domain [83]. The protein product interacts with proline-tyrosine motif–containing proteins mainly via its WW1 domain [84, 85]. Through these interactions, WWOX regulates many signaling molecules, leading to changes in gene expression, protein stability, and subcellular localization that underlie its pleiotropic functions in cell survival, proliferation, differentiation, autophagy, and metabolism (reviewed in [86–89]). In line with its involvement in this broad spectrum of essential biological functions, *Wwox* knockout mice display complex metabolic and neurological phenotypes and die by two to three weeks of age [90, 91].

**Fig 3:**
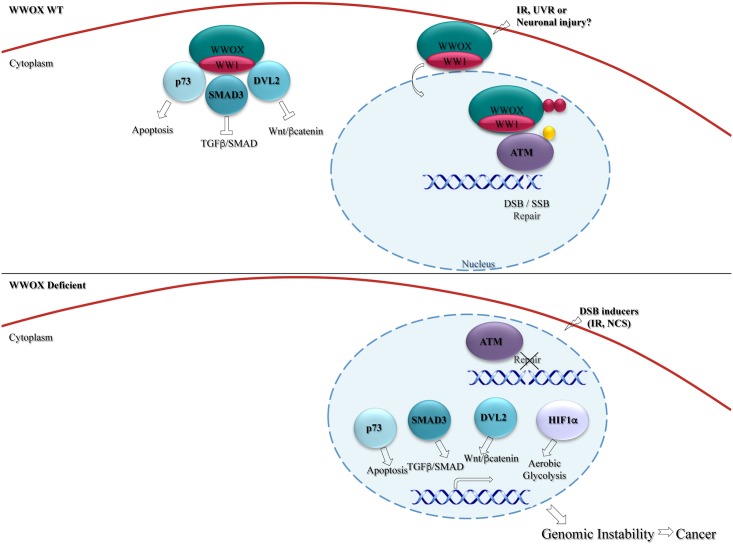
Tumor suppressive functions of WWOX. WWOX interacts with signaling molecules in the cytoplasm and prevents their entry to the nucleus to modulate their tumorigenic pathways. Upon stress such as ionizing radiation (IR), ultraviolet radiation (UV), or maybe even neuronal injury, WWOX enters the nucleus and promotes DSB repair. In the absence of WWOX, DSB repair is impaired and WWOX-interacting molecules enter the nucleus and induce their tumorigenic signals.

Like FHIT, WWOX has a range of tumor-suppressing functions, affecting DNA repair and apoptosis, which identifies it as a master regulator that protects cells against genomic instability and tumor progression (Fig 3). In response to DSBs, WWOX levels increase, and this is associated with activation of ATM, promoting efficient and accurate DNA repair [92]. ATM activation enhances activity of the ubiquitin E3 ligase ITCH [93, 94] as well as other substrates including H2A.X, CHK2, and p53 [95, 96]. In a feed-forward loop, ITCH mediates K63-linked ubiquitination of WWOX and promotes its translocation into the nucleus [92]. Nuclear WWOX interacts with ATM, enhancing ATM monomerization and activation. Targeted WWOX depletion reduces ATM activation, decreases phosphorylation of H2A.X and CHK2, and results in defects in recruitment of checkpoint proteins to DNA-damage sites [92]. Consistent with these observations, overexpression of WWOX increases the efficiency of homologous recombination (HR) repair [92].

Recent findings have also revealed that WWOX levels accumulate in response to DNA SSBs [97]. After SSBs, WWOX binds p-ATM and modulates the ATM checkpoint activation as well as the ATR signaling pathway [97]. Since ATM inhibition is associated with reduced ATR activation, WWOX may modulate ATR function in an ATM-dependent manner (Fig 3). ATM inhibition also results in several changes that could affect DDR signaling; for example, it increases levels of the tumor suppressor ARF [98, 99]. Hence, the regulatory link between WWOX and ATM raises a potential involvement of WWOX in regulating other players in this signaling pathway, including ARF. Loss of WWOX results in delayed activation of the DNA damage checkpoint kinase ATM and impaired DNA repair, rendering the genome less stable (Fig 3) [24]. Indeed, exposure of *Wwox*-knockout–derived mouse embryonic fibroblasts to aphidicolin is associated with increased chromosomal breaks, a phenotype that is rescued upon restoration of wild-type WWOX [97].

Another role for WWOX in DDR is the induction of apoptosis through interactions with the pro-apoptotic transcription factors of the p53 family: p53, p73, and p63. Upon DNA damage, WWOX interacts with and stabilizes these factors, leading to apoptosis through various mechanisms that are mostly unclear. Interaction between WWOX and p73 in the nucleus and cytoplasm leads to transactivation-independent apoptosis (Fig 3) [84, 100, 101]. WWOX also specifically binds ΔNp63α but not TAp63 [102]. This interaction stabilizes ΔNp63α, inhibiting its translocation to the nucleus and suppressing its transactivation function. The interaction between WWOX and PPxY-containing proteins, including p73 and ΔNp63α, occurs through its WW1 domain. Interestingly, the Chang group has shown that when murine WOX1 is phosphorylated on Tyr33, it binds p53 and synergistically enhances p53-mediated apoptosis [103, 104]. Thus, distinct regions of WWOX interact with different members of the p53 family to regulate apoptosis.

Another emerging function of WWOX is in maintaining homeostasis of the nervous system (reviewed in [89, 105]). Loss of WWOX in animal models is associated with epilepsy and ataxia [106, 107]. In agreement with these observations, several recent reports have described homozygous mutation of *WWOX* in epileptic patients [108–110]. Some of these reports described early onset death of patients, as shown in the *Wwox* null mice [17, 90, 91], precluding adult tumor analysis. Other reports described adult epileptic patients homozygous for mutant WWOX but with no sign of tumor development, suggesting that other hits and/or mutations might be required for cancer to develop [87, 105]. It remains unknown whether these neuronal defects result from impaired DDR function of WWOX or whether patients harboring *WWOX* null mutations are more sensitive to tumor development over their life span.

Cumulatively, these observations indicate that the gene product of FRA16D, WWOX, behaves as a tumor suppressor by affecting several signaling pathways and promoting efficient DDR.

### PARKIN/FRA6E

FRA6E spans a region of about 3.6 Mb at 6q25–q27, which contains eight genes. One of these genes is the Parkin (*PARK2*), which encodes an ubiquitin E3 ligase [111]. PARK2 contains a ubiquitin-like domain at its N-terminus and two RING finger motifs and an In Between Ring fingers (IBR) domain at its C-terminus. PARK2 transcripts are reduced or absent in many cancer types [112–115]. Genome analysis of 5,000 individual tumors comprising 11 tumor types showed that PARK2 is frequently deleted in human cancers. It also uncovered a striking pattern of mutual exclusivity between PARK2 deletion and amplification of cyclin D1 (*CCND1*), cyclin E1 (*CCNE1*), or cyclin-dependent kinase 4 (*CDK4*), implicating these genes in a common pathway. Consistent with this, PARK2 was found to regulate coordinated degradation of these G1/S cyclins [116], while inactivation of *PARK2* resulted in cyclin D1 accumulation and acceleration of cell cycle progression. In a recent report, it was shown that PARK2 regulates mitosis and genomic stability through targeting the anaphase promoting complex/cyclosome (APC/C) [117]. Loss of PARK2 causes increased levels of mitosis regulators that may contribute to various forms of chromosome instability and may cause tumor formation [117].

PARK2 was also identified as a p53 target that mediates p53 tumor suppression functions in energy metabolism and antioxidant defense [118]. It modulates PARK7 (DJ-1) transcription and protein levels via a signaling cascade involving p53 and the endoplasmic reticulum (ER) stress–induced active X-box–binding protein-1S (XBP-1S). In a negative feedback loop, PARK2 triggers transcriptional repression of p53 while p53 down-regulates expression of PARK7 protein and mRNA [119].

Interestingly, PARK2, like WWOX, is implicated in a neurodegenerative disorder; Parkinson’s disease (PD) [120]. While somatic mutations of PARK2 that decrease PARK2’s E3 ligase activity, compromising its ability to ubiquitinate cyclin E, are associated with cancer [121, 122], germline mutations of PARK2 were identified in patients with autosomal recessive juvenile Parkinsonism (ARJP) [123]. One of the main causes of PD is a redox imbalance in the brain [124]. Postmortem studies of PD patients reveal increased oxidation of lipids, proteins, and DNA, a severe decrease in glutathione concentration, and an accumulation of superoxide dismutase (SOD2) (reviewed in [125]). Oxidative DNA damage occurs to a higher extent in PD individuals compared with age-matched controls [126]. It is not known, however, whether this DNA damage selectively targets FRA6E as a hot spot harboring PARK2 or might also target other fragile sites and genes (i.e., WWOX).

In summary, PARK2, a gene product of FRA6E, is a tightly regulated protein that is implicated in tumor suppression and neuropathy.

### SPIDR/FRA8I

Another CFS-associated gene product with a known function in the DDR is scaffolding protein involved in DNA repair (SPIDR)/KIAA0146. The fragile site FRA8I is unstable in lymphocytes and epithelial cells and is disrupted frequently in colorectal cancer [127]. FRA8I spans 530 kb at 8q11.21 and encompasses the regions encoding SPIDR, CEBPD, and part of PRKDC. The latter two genes encode proteins involved in tumorigenesis in a variety of cancers. SPIDR is a translocation partner of the immunoglobulin heavy chain gene in recurrent t(8;14)(q11;q32) translocations in a subset of patients with B cell precursor acute lymphoblastic leukemia [128].

Recent evidence has shown that SPIDR plays a critical role in DSB repair by regulating HR [129]. As a scaffolding protein, SPIDR promotes the recruitment of DNA-processing enzymes like the helicase BLM and recombinase RAD51 to sites of DNA damage and thereby contributes to maintenance of genomic integrity [129]. SPIDR was also found to interact with RAD51-binding protein fidgetin-like 1 (FIGNL1) to mediate its function in HR [130]. Depletion of SPIDR results in an increased rate of sister chromatid exchange, defects in HR, hypersensitivity to DNA-damaging agents, and overall genome instability. Knockout mouse studies of SPIDR/KIAA0146 are currently unavailable, but functional consequences of its targeted loss should be important to pursue in the future.

### RORA/FRA15A

The orphan retinoic receptor alpha (*RORA*) gene is encoded in the middle of the FRA15A region at chromosome 15q22. RORA is expressed in normal breast, prostate, and ovarian epithelium tissues but is frequently inactivated in cancers that arise from these organs [131–134]. In addition, RORA is involved in other pathophysiological processes such as cerebellar ataxia, inflammation, atherosclerosis, and angiogenesis [135]. RORA expression is induced in response to aphidicolin treatment, and its loss correlates with fragility of FRA15A [134]. The RORA transcript is also induced upon exposure to UV radiation, H_2_O_2_, and methyl methanesulfonate, suggesting a role in a broad spectrum rather than a specific DNA lesion. Nevertheless, the exact molecular function of RORA in the DDR remains to be identified.

### Conclusions and Future Perspectives

The prevailing view of CFS biology in the literature has associated their expression with occurrence of uncontrolled breaks or gaps in metaphase chromosomes and their association with human pathologies (mainly cancer). This view has been recently challenged by revealing the existence of an active mechanism that is responsible for controlled generation of DNA DSBs at CFSs and the evidence that this process enhances, rather than compromises, the maintenance of genome integrity [136]. This emerging evidence and the fact that CFS loci are conserved, harboring coding and noncoding genes that are actively (at least some) engaged in vital cellular processes including apoptosis and DNA repair challenges the prevailing view of CFSs being inert passive structures.

**Fig 4:**
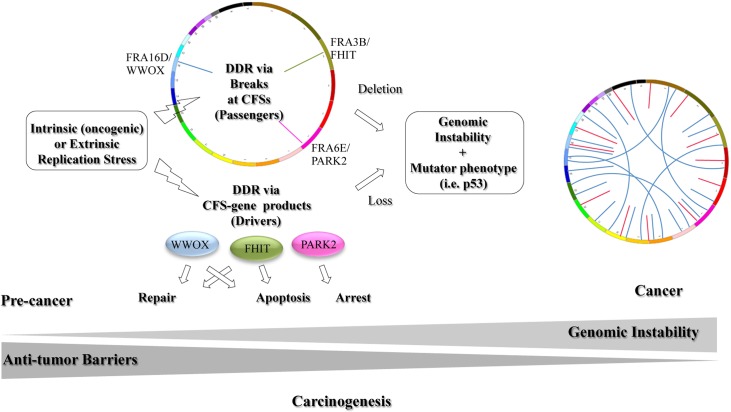
Hierarchical model of cancer progression links between replication stress, DDR, CFS-Gs, and genomic instability. CFSs and their gene product are affected by replication stress. While CFSs (for example, FRA3B, FRA16D, and FRA6E) are prone to DSBs, CFS-Ps (FHIT, WWOX, PARK2) modulate DNA damage repair and/or apoptosis. Upon extensive damage, breaks within CFSs appears as deletions. Cells with deletions within CFS-Gs are positively selected because of their roles in DDR, leading to genomic instability and a mutator phenotype. Additional mutations in classical recessive genes (i.e., *Trp53*) would release the antitumor barriers (apoptosis and senescence), leading to cancer progression.

The association of CFS loci and genomic instability in cancer has been claimed for decades [137, 138]; however, the tumor suppressive functions of some of the CFS-Ps were only recently revealed. The studies reviewed here strongly support the identity of at least some CFSs as important functional units involved in tumor suppression through effects on the DDR and other signaling pathways and suggests that their loss during carcinogenesis promotes genome instability. The emerging functions of CFS-Ps in the DDR and additional tumor suppressive pathways raise the possibility that instability of CFSs appear early in precancerous lesions not only because of their fragility but also since their deletion is selected by growth advantage following loss of their tumor suppressor functions (Fig 4). The existing evidence suggests that CFS-Gs secure the safety of the cells by promoting the DDR following DNA damage, resulting in apoptosis, cell cycle arrest, or DNA repair (reviewed in [23–25]. When CFS-Gs are lost and the DDR effectors such as p53 are mutated, the circuit becomes unregulated—proliferation proceeds and the cells acquire a selective and clonal growth advantage.

In agreement with a previous hypothesis [139], we propose that the sensitivity of a given CFS in a specific cell type could function as a warning sensor for DNA damage that activates the classical DDR (i.e., ATR and/or ATM signaling). According to this hypothesis, CFSs are scattered throughout the genome in various chromosomes to protect against detrimental effects from DNA damage and replication stress and to activate the DDR and maintain the integrity of the genome. At the same time, mounting evidence show that CFS-Ps become activated to maintain proper DDR. According to our model, CFS tumor suppressor genes act to facilitate or maintain the cellular functions of the classical DDR. For example, they may support the canonical DDR checkpoint proteins, as in the case of WWOX, FHIT, and PARK2 (Fig 4). Indeed, numerous studies show that some CFS-Ps have clearly important roles in the DDR, as illustrated first by their induction in response to various DNA-damaging agents and second by the genomic instability and accumulation of mutations associated with their loss, as seen in mouse models and in human cell lines (reviewed in [23–25]).

It remains a mystery why CFSs, the most unstable chromosomal regions in the genome, are evolutionarily conserved both in organization and structure (sequence level) and frequently harbor tumor-suppressor genes. One potential explanation is that many cancer types occur relatively late in life when the reproductive phase is finished. In support of this assumption, germline mutations or loss of function of *WWOX*, *RORA*, and *PARK2* are associated with neurological diseases, whereas somatic deletions are associated with cancer.

Even though loss of CFS-Gs can be a prominent lead of tumorigenesis, it also has the potential to be a target for cancer therapy. CFS-Gs may offer two possible applications: the use of their loss as (1) biomarkers for early molecular diagnostic tools and (2) as synthetic lethality hits to discover antitumour drugs that sensitize tumor cells to undergo apoptosis.

While affirming the importance of some of the CFS-Ps in DDR and their involvement in cancer, the observations reviewed here also raise new questions such as: Why do different tissues and cancer cells respond so differently to replication stress and expression of CFSs? What is the molecular basis of other less classified CFS-Ps activation and behavior in DDR and in cancer? Do the impaired functions of CFS-Ps in neurological disorders involve their role in the DDR? Are CFS-Ps part of a global stress response network, and is deletion of their encoded genes mutually exclusive or concordant in the same cancer sample? Does alteration at CFS loci play a role in diseases other than cancer? Addressing these questions and others will be facilitated by pan-studies mapping all CFSs in different cell types and contexts and deciphering their roles in different disease states. Using advanced and emerging next-generation sequencing methods and state-of-the-art technologies [140] should allow such future studies and better understanding of functional consequences of CFSs’ alteration, their role in cancer development, and their likely role in other disorders such as neurodegenerative and metabolic diseases.
